# Congenital Upper Limb Deficiency with Oligodactyly: A Case Report

**DOI:** 10.31729/jnma.7585

**Published:** 2022-08-31

**Authors:** Anu Radha Twayana, Neela Sunuwar, Sulav Deo, Sushil Rayamajhi, Ninda Tashi Tenzing Sherpa, Firoz Anjum

**Affiliations:** 1 Department of Medicine, Bhaktapur Hospital, Dudhpati-1, Bhaktapur, Nepal; 2 B. P. Koirala Institute of Health Sciences, Dharan, Sunsari, Nepal; 3 Department of Medicine, Suraksha Hospital, Biratnagar-7, Morang, Nepal; 4 Department of Medicine, Swacon International Hospital, Battisputali, Kathmandu, Nepal; 5 Kathmandu University School of Medical Sciences, Dhulikhel, Kavre, Nepal; 6 Department of Paediatrics, Patan Academy of Health Sciences, Lagankhel, Lalitpur, Nepal

**Keywords:** *congenital limb deficiency*, *prevalence*, *upper limb defect*

## Abstract

Congenital upper limb deficiency care and management have undergone drastic changes over the past 50 years. Given the low incidence of this defect nationwide, this case report adds to the existing database to analyze the etiological investigations, descriptive epidemiology, and trend detection. We present a case of a 2-month-old male child with congenital upper limb deficiency and explore the possible etiologies and difficulties during early diagnosis of such rare disorders in a low and middle-income countrries and present implications at the primary health care level to improve the prognosis. Poor nutrition during pregnancy due to an unplanned pregnancy is a possible cause. The prognosis is unfavourable due to sociocultural barriers. To address these limitations, it is pertinent to address disability-adequate knowledge among communities, promote early diagnosis, and timely rehabilitation using a multidisciplinary approach. Further, we provide a framework to optimize care.

## INTRODUCTION

Over the past 50 years, the clinical management of congenital upper limb deficiency has been drastically changed.^1^ The incidence of upper limb deficits was 3.4 per 10,000 live births according to a study done in Nepal.^2^ The aetiology is complex.^1^ We present a case of a 2-month-old male child with congenital limb deficiency and explore the possible etiologies. We further discuss the difficulties during the early diagnosis of such rare congenital disorders in Low- or Middle-Income countries (LMIC) like Nepal and present implications at the primary health care level to improve the prognosis.

## CASE REPORT

**Figure 1:**
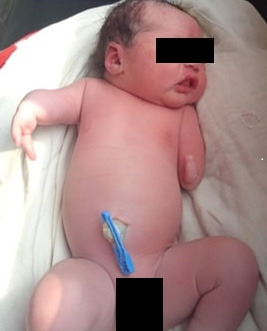
Depiction of the child with congenital upper limb deficiency.

A 2-month-old male child was born to Gravida 2, Para 1, and Abortion 1 (G2P1A1) at 39 weeks of gestation by cesarean section due to cephalo-pelvic disproportion. There was no history of consanguinity, and the prenatal course was uneventful. All prenatal ultrasounds were reported as "normal". The mother took iron and calcium tablets but no folic acid during the course of the pregnancy. She had no history of infection during pregnancy. There was no history of prenatal exposure to radiation or the consumption of any teratogenic drugs during the prenatal period. The cesarean delivery was uncomplicated. The child weighed 4.2 kgs at birth. The baby cried immediately after birth. His Apgar scores were 8/10 and 9/10 at 1 and 5 minutes, respectively. On physical examination, the head and neck were normal. However, it was noted that the baby had short upper limbs with only two digits on the left hand and an absent left forearm. In addition, the right forearm was short with two digits attached to the distal forearm (Figure 1).

On further examination, his anthropometry was within the normal limit and he could move all his digits and limbs. All other systemic examination findings were unremarkable. Exclusive breastfeeding was started immediately after birth and he passed urine and meconium within the first 24 hours of life.

**Figure 2:**
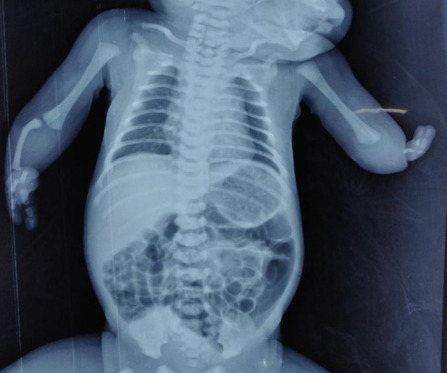
X-ray image of the child with congenital upper limb deficiency.

The mother had no prior history of tobacco use or alcohol consumption. Furthermore, diabetes or hypertension was not documented prior to or during the course of the pregnancy. She had a history of spontaneous abortion at 8 weeks of gestational age a year back. There was no family history of similar defects. The baby's X-ray report suggested a type IV radial longitudinal defect in the right forearm and an intercalary defect in the left arm or distal phocomelia with two digits directly attached to the humerus (Figure 2).

To rule out an association with other congenital diseases like vertebral defects, anal atresia, cardiac defects, tracheo-oesophageal fistula, renal anomalies, and limb abnormalities (VACTERL) association, Fanconi anaemia, and genetic syndromes like Holt-Oram Syndrome, a complete blood count panel, echocardiography, and ultrasonography (USG) abdomen were ordered and were found to be within the normal range. Other pertinent investigations could not be carried out owing to resource limitations. The baby and the mother were observed for a week and were discharged after proper genetic counselling in consultation with the pediatric neonatologist. The baby was advised of regular follow-ups and was referred to the orthopaedics department for further management and rehabilitation.

## DISCUSSION

The phenotypic symptoms of congenital limb malformations are diverse.^3,4^ Congenital limb malformations are defined as (I) failure of part formation, (II) failure of part differentiation (separation), (III) duplication, (IV) overgrowth, (V) undergrowth, (VI) congenital constriction band syndrome, and (VII) generalized skeletal abnormalities, according to the International Federation of Societies for Surgery of the Hand and the International Society of Prosthetics and Orthotics.^1^ Congenital abnormality affects between 1 and 2% of live births. Of these, around 10% have upper-limb deformities.^5^ A study conducted in Nepal, in 2015 pointed out that although genetic and environmental factors are major contributors to birth defects in Nepal, poor nutrition, low maternal body mass index (BMI), unplanned pregnancy, folic acid supplementation late in pregnancy, and general lack of awareness are causative of birth defects.^6^ In the case of unintended pregnancies, mothers typically seek medical help after the first trimester or not at all. The child of an unplanned conception is at greater risk of abortion, being underweight at birth, dying in the first year of life, being abused, and receiving insufficient resources for healthy development.^7^ In our case, the mother had a prior abortion pointing to the consequence of an unplanned pregnancy. Consequently, the unborn and newborn children were at an added risk. A study done in China found that folic acid supplementation leads to a reduction in limb defects. The mother in our case also had poor nutrition and did not take folic acid supplements, thus adding value to this study's findings.^8^

A one-year review study from 2003-2004 done in Maternity Hospital (Prasuti Griha), Nepal found 14 cases of musculoskeletal anomalies upon deliveries. The study further concluded that congenital defects of both the upper (3.4/10000) and lower limbs (1.1/10000) may occur mainly due to teratogen exposure during the 4th-5th weeks of gestation.^2^ Infectious agents like toxoplasmosis, rubella, cytomegalovirus, herpes, and human immunodeficiency virus (HIV) virus (TORCH) during embryogenesis play a vital role; however, the use of the Rubella vaccine has reduced the risks of major congenital anomalies by 85%.^2^ In contrast to contributing factors of congenital limb defects, the mother in our case did not take any teratogenic medications and had no pre or postnatal infections and was vaccinated as per guidelines led out by the Nepalese government. Thus, poor nutrition and an unplanned pregnancy is a possible hypothesis for the baby's congenital limb defect. A central limitation to tying in these findings was that we could not carry out molecular and/or genetic investigations because of inadequate resources in the LMIC setting. As per one study, congenital limb defects require early diagnosis and prompt rehabilitation to gain optimum limb function while improving the cosmetic appearance.^9^ The medical and financial costs of congenital abnormalities are enormous.^10^ A thorough assessment is henceforth necessary to promote age-specific mental and physical development. The treatment is multidisciplinary, requiring reconstruction of digits while preserving sensory and motor functionality. Further, fitting prosthetics for cosmetic purposes along with long-term occupational therapy and physiotherapy ought to be the primary care consideration in similar cases. In our child, we made referrals to the orthopaedic surgeon team, to probe for timely surgical intervention and genetic counselling.

In the context of early diagnosis and prompt rehabilitation, Nepal lags behind in speciality services, especially in the rural setting where individuals, particularly pregnant females, are unaware of congenital anomalies or internalize them as a consequence of their wrongdoings. This is a pertinent socio-cultural issue as mothers link their child's congenital limb defects to their sins, with other religious themes. Children are often socially ostracized and deprived of basic needs in the name of faith and religion. Comprehensive fetal ultrasonography enables an early prenatal diagnosis of congenital limb defects and offers critical information to the parents, assisting them in making decisions about the pregnancy's fate.^11^ As illustrated in the preliminary framework to optimize care in LMICs, it is necessary to promote awareness using public health messages, pursue action plans for improved surveillance, prevention of anomalies using improved diagnostic testing facilities, research trends in the country, and optimize care for children affected by birth defects.

Randomized trials and several observational studies have indicated that folic acid supplementation during pregnancy, either alone or in combination with other vitamins, is beneficial in preventing neural tube abnormalities. The question of whether this holds true for other congenital defects is a complicated one, and it is the subject of this study. It's vital to assess the data not just for individual birth problems, but also for all birth defects taken together.^12^ Similarly, it is important to consider ethnicity to the incidence of congenital abnormality due to economic inequalities that may modify exposures; advantageous ethnic groups like Brahmin and Chettri are comparatively less likely to be connected with birth abnormalities than non-advantageous ethnic groups and this was supported by the findings of a multicenter study done in Nepal.^13^

The age of the mother also should be considered because of the known link between maternal age and poor pregnancy outcomes. Furthermore, given the known association between gastroschisis and amelia in newborns of younger mothers, it's likely that a vascular disruption is at the root of the congenital limb abnormality.

Congenital limb deficiency is a rare genetic disorder. Besides genetic and chromosomal abnormalities, poor nutrition during pregnancy as a consequence of unplanned pregnancy could be one of the contributory factors. To address these limitations, it is important to take into consideration various reasons for a disability such as lack of adequate knowledge among communities, promoting early diagnosis, and timely rehabilitation using a multidisciplinary approach and informed framework. One way is to have a birth defects surveillance system. The birth defects surveillance system contains a large database of infants with major and minor exterior structural malformations, as well as the unique characteristic of a photographic record in the majority of instances. These data can be used for etiological investigations, descriptive epidemiology, and trend detection.

## References

[1] Oberg KC (2019). Classification of congenital upper limb anomalies: towards improved communication, diagnosis, and discovery.. J Hand Surg Eur Vol..

[2] Malla BK (2007). One year review study of congenital anatomical malformation at birth in Maternity Hospital (Prasutigriha), Thapathali, Kathmandu.. Kathmandu Univ Med J (KUMJ)..

[3] Koskimies E, Lindfors N, Gissler M, Peltonen J, Nietosvaara Y (2011). Congenital upper limb deficiencies and associated malformations in Finland: a population-based study.. J Hand Surg Am..

[4] Baas M, Zwanenburg PR, Hovius SER, van Nieuwenhoven CA (2018). Documenting Combined Congenital Upper Limb Anomalies Using the Oberg, Manske, and Tonkin Classification: Implications for Epidemiological Research and Outcome Comparisons.. J Hand Surg Am..

[5] Duteille F, Beneteau C, Camut MV, Perrot P (2016). Congenital deformities of the hand and upper limb.. Ann Chir Plast Esthet..

[6] Bhandari S, Sayami JT, KC RR, Banjara MR (2015). Prevalence of congenital defects including selected neural tube defects in Nepal: results from a health survey.. BMC Pediatr.

[7] Luchters S, Bosire W, Feng A, Richter ML, King'ola N, Ampt F (2016). "A baby was an added burden": predictors and consequences of unintended pregnancies for female sex workers in Mombasa, Kenya: A Mixed-Methods Study.. PLoS One.

[8] Liu J, Li Z, Ye R, Ren A, Liu J (2019). Folic acid supplementation and risk for congenital limb reduction defects in China.. Int J Epidemiol..

[9] Watson S (2000). The principles of management of congenital anomalies of the upper limb.. Arch Dis Child..

[10] Ingrid Goh Y, Bollano E, Einarson TR, Koren G (2006). Prenatal multivitamin supplementation and rates of congenital anomalies: a meta-analysis.. J Obstet Gynaecol Can..

[11] Makhoul IR, Goldstein I, Smolkin T, Avrahami R, Sujov P (2003). Congenital limb deficiencies in newborn infants: prevalence, characteristics and prenatal diagnosis.. Prenat Diagn..

[12] Botto LD, Olney RS, Erickson JD (2004). Vitamin supplements and the risk for congenital anomalies other than neural tube defects.. Am J Med Genet C Semin Med Genet..

[13] Paudel P, Sunny AK, Gurung R, Gurung A, Malla H, Rana NB (2021). Burden and consequence of birth defects in Nepal-evidence from prospective cohort study.. BMC Pediatr..

